# Clinical Outcome of Hypertensive Uveitis

**DOI:** 10.1155/2015/974870

**Published:** 2015-10-04

**Authors:** Deborah Lewkowicz, François Willermain, Lia Judice Relvas, Dorine Makhoul, Sarah Janssens, Xavier Janssens, Laure Caspers

**Affiliations:** ^1^CHU Saint-Pierre, Université Libre de Bruxelles (ULB), 1000 Brussels, Belgium; ^2^CHU Brugmann, Université Libre de Bruxelles (ULB), 1000 Brussels, Belgium

## Abstract

*Purpose*. To review the clinical outcome of patients with hypertensive uveitis. *Methods*. Retrospective review of uveitis patients with elevated intraocular pressure (IOP) > 25 mmHg and >1-year follow-up. Data are uveitis type, etiology, viral (VU) and nonviral uveitis (NVU), IOP, and medical and/or surgical treatment. *Results*. In 61 patients, IOP values are first 32.9 mmHg (SD: 9.0), highest 36.6 mmHg (SD: 9.9), 3 months after the first episode 19.54 mmHg (SD: 9.16), and end of follow-up 15.5 mmHg (SD: 6.24). Patients with VU (*n* = 25) were older (50.6 y/35.7 y, *p* = 0.014) and had more unilateral disease (100%/72.22%  *p* = 0.004) than those with NVU (*n* = 36). Thirty patients (49.2%) had an elevated IOP before topical corticosteroid treatment. Patients with viral uveitis might have higher first elevated IOP (36.0/27.5 mmHg, *p* = 0,008) and maximal IOP (40.28/34.06 mmHg, *p* = 0.0148) but this was not significant when limited to the measurements before the use of topical corticosteroids (*p* = 0.260 and 0.160). Glaucoma occurred in 15 patients (24.59%) and was suspected in 11 (18.03%) without difference in viral and nonviral groups (*p* = 0.774). *Conclusion*. Patients with VU were older and had more unilateral hypertensive uveitis. Glaucoma frequently complicates hypertensive uveitis. Half of the patients had an elevated IOP before topical corticosteroid treatment.

## 1. Introduction

Elevated intraocular pressure (IOP) is a frequent complication of intraocular inflammation, affecting 5 to 19% of uveitis patients [1]. Elevated IOP can be acute or chronic and both presentations can lead to optic nerve damage and visual field defect in glaucoma secondary to hypertensive uveitis. IOP elevation may have different origins: trabeculitis, obstruction of the trabecular meshwork, pupillary block due to posterior synechiae, or steroid induced [1]. The management of hypertensive uveitis is very difficult. The treatment begins classically with the use of topical hypotensive drugs like beta-blockers, alpha-agonist, and carbonic anhydrase inhibitors. Although it has been reported in the past that prostaglandins analogs could induce uveitis, more recent studies have found their relative safety in nonviral uveitis, and they are thus now also used for the management of intraocular hypertension in some uveitis patients [2–4]. When treatment with topical drops fails to normalize intraocular pressure, oral acetazolamide can also be added but is often not well tolerated for long term treatment. When medical treatment is no longer sufficient or requires chronic oral acetazolamide, a surgical approach is usually proposed [3–5].

Elevated IOP and glaucoma have been described more frequently in certain uveitis entities, such as juvenile rheumatoid arthritis, sarcoidosis, Vogt-Koyanagi-Harada syndrome, sympathetic ophthalmia, syphilis, or toxoplasmosis. Viral uveitis, including rubella virus (RV) infection associated with clinical diagnosis of Fuchs uveitis and anterior uveitis due to herpes virus infection (herpes simplex virus (HSV), herpes zoster virus (VZV), and cytomegalovirus (CMV)), is another uveitis group also frequently associated with elevated IOP and glaucoma [5–9]. Few studies have compared viral and nonviral hypertensive uveitis in terms of IOP characteristics or evolution towards glaucoma [1, 7, 10, 11]. Therefore we retrospectively reviewed the clinical outcome of our series of uveitis patients with elevated IOP and analyzed their outcome and compared patients with viral and nonviral uveitis.

## 2. Materials and Methods

We retrospectively retrieved the medical records of consecutive patients with uveitis and IOP higher than 25 mmHg at the Department of Ophthalmology in the CHU Saint-Pierre, Brussels, Belgium, who presented to our department since the first episode of uveitis between 2003 and June 2012. This study was accepted by the ethic committee of the hospital. In order to obtain a complete and long enough follow-up of patients since the first episode of hypertensive uveitis, all patients already treated for uveitis before their referral to our department or who did not have a minimum of 1-year follow-up were excluded from the study. Patients with closed angle glaucoma and pupillary seclusion were also excluded from the study. Clinical data were collected including results from systemic work-up, etiology, first IOP at presentation of uveitis, first elevated IOP, time before elevation of intraocular pressure, maximal IOP, IOP at 3 months, at 1 year, and at last visit, need for surgery, and occurrence of glaucoma as defined by Casson et al. [12]. The diagnosis of glaucoma was based on visual field defects measured by the automated visual field Humphrey (test 24-2) and optic disc analysis of pathologic cupping of the optic nerve head (inferior thinning of nerve fiber layers during quiescent periods of uveitis) analyzed by OCT Zeiss Spectralis in patients with elevated IOP based on the diagnostic tools for glaucoma detection and management reported by Sharma et al. [13]. A suspected glaucoma was defined as glaucomatous defects on visual field or optic nerve head detected by OCT but not both tests together in patients with elevated IOP [13]. Elevated IOP with no glaucoma corresponded to a lack of glaucomatous defects on visual field and optic disc OCT in these patients. All patients included had an appropriated work-up depending on the type of uveitis with at least syphilis serology, chest X-ray, and Mantoux test for granulomatous uveitis and HLA B27 test for nongranulomatous uveitis. Diagnoses of specific uveitis entities, such as birdshot, Vogt-Koyanagi-Harada (VKH), and sarcoidosis, were based on SUN criteria and specific criteria [14–16]. Diagnosis of viral uveitis was based on clinical characteristic and/or positive polymerase chain reaction (PCR) for a virus. For the diagnosis of CMV anterior uveitis, selection of patients was based on the association of anterior uveitis with a few large precipitates surrounded by endothelial inflammation (coin shaped) and positive PCR for CMV in all the cases [17]. A positive PCR for HSV1, HSV2, or VZV was used in most cases to support the diagnosis. Diagnosis of Fuchs heterochromic uveitis was based on pathognomonic spindle shape keratic precipitates scattered all over the cornea with diffuse iris changes including heterochromia, depigmentation, and velvet aspect of the iris surface [6, 18]. Fuchs heterochromic uveitis has been recognized as a viral uveitis. Rubella virus has been mostly implicated but CMV might also be implicated in Fuchs heterochromic uveitis [6–9]. Therefore, PCR for CMV and rubella have also been performed in some of these patients. For the diagnosis of herpetic anterior uveitis, the diagnosis was based on clinical characteristics including typical sectorial iris atrophy pathognomonic for herpetic anterior uveitis and associated scars of stromal keratitis and endotheliitis or previous zoster ophthalmicus in the frontal and nasal area [19, 20].

In order to perform a statistical analysis, when both eyes had hypertensive uveitis, only the worse eye was selected for the study based on the worse evolution of the hypertony IOP most uncontrolled or most topically and/or orally (acetazolamide) treated to control IOP. Patients with viral uveitis were compared to patients with nonviral uveitis.

The appropriate tests used for statistical analysis are detailed in the tables for each statistical analysis. *p* values < 0.05 were considered to be statistically significant.

## 3. Results

We collected 61 consecutive patients with hypertensive uveitis. Ten patients had a bilateral hypertensive uveitis but for statistical analysis we only included in the study 61 eyes with the most severe HIOP. We found 25 patients with viral uveitis including 18 patients with positive PCR for a virus (5: Fuchs (2 PCR+), 14: herpes (10 PCR+), and 6: CMV (6 PCR+)) and 36 nonviral uveitis including 6: toxoplasmosis, 3: Behçet, 3: Vogt-Koyanagi-Harada (VKH), 7: sarcoidosis (1 defined, 2 presumed, and 4 probable as defined by the international criteria for the diagnosis of ocular sarcoidosis: results of the first International Workshop On Ocular Sarcoidosis) [15], 1: juvenile idiopathic arthritis (JIA), 2: ankylosing spondylitis (SPA), and 14: idiopathic (Table 1).

The number of patients analyzed in the viral group did not differ statistically from nonviral group (25 and 36, *p* = 0.159). Patients were significantly older in viral group than in the nonviral group (50.6 and 35.7 years, *p* = 0.014). No difference was found in the M/F ratio in both viral and nonviral groups (13/12 and 19/17, *p* = 1.0). The mean follow-up duration was 67.8 months (range 6–205 months, SD: 37.8) in the whole series of patients with no significant difference between viral and nonviral groups (65.9 and 69.1 months, *p* = 0.743) (Table 2).

All patients from the viral group had unilateral uveitis while bilateral hypertensive uveitis was observed in 10 patients (16.4%) of the nonviral group. This difference was statistically significant (*p* = 0.004) (Table 3).

Mean time before first episode of elevated IOP was 9.3 months (range 0–141, SD: 23.0). There was no significant difference in time to first episode of elevated IOP (*p* = 0.080) between viral and nonviral group of uveitis and Table 3.

**Figure 1:**
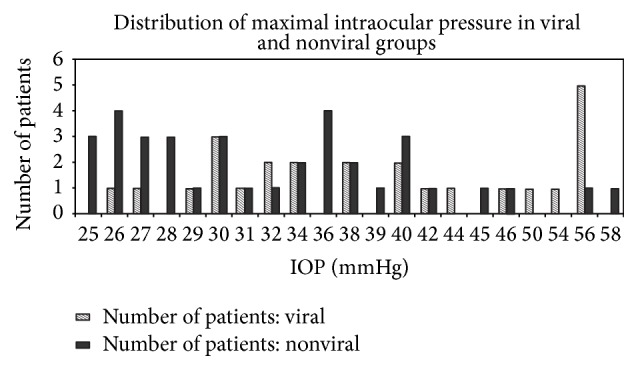


The mean value of the first elevated IOP in the whole series of patients was 32.9 mmHg (range 25–58, SD: 9.0) which was significantly higher in the viral group (36.0 mmHg) than in the nonviral group (27.5 mmHg) (*p* = 0.008) (Table 3). The highest (maximal) values of IOP are detailed in Figure 1. The mean highest value of IOP for the whole group of patients was 36.6 mmHg (range 25–58, SD: 9.9). The mean highest value of IOP was significantly higher (40.3 mmHg, range 26–56, SD: 10.6) in the viral group than in the nonviral group (34.1 mmHg, range 25–58, SD: 8.7) (*p* = 0.015) (Table 3). Thirty patients (49.20%) (15 in the viral group and 15 in the nonviral group) had elevated IOP before applying corticosteroid drops. Among these 30 patients there was no significant difference in first or maximal elevated IOP in viral and nonviral group (*p* = 0.260 and 0.160).

**Figure 2:**
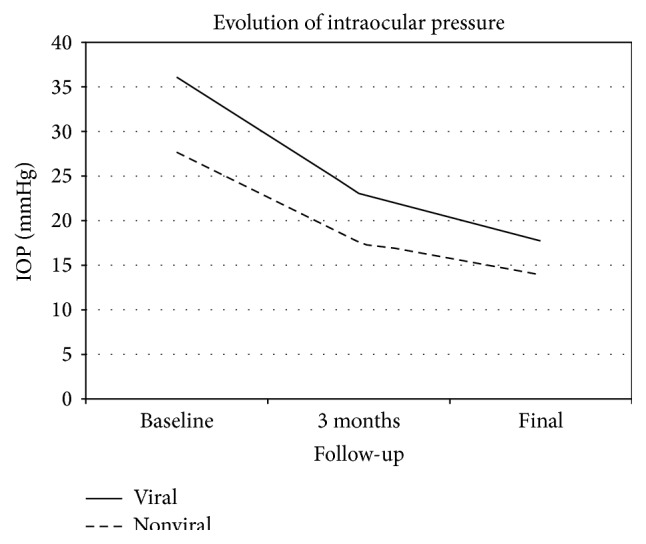


At 3 months after the first episode, the mean IOP values dropped to 19.54 mmHg (range 8–56, SD: 9.16) in the whole group with IOP of 23.0 mmHg (range 10–56, SD: 11.4) in the viral group and 17.5 mmHg (range 8–48, SD: 6.3) in the nonviral group (*p* = 0.057) (Figure 2). At the end of the follow-up we found a mean IOP of 15.5 mmHg in the whole series of patients (range 6–47, SD: 6.24) with a mean IOP of 17.7 mmHg (range 10–47, SD: 8.3) in the viral group and 13.94 mmHg (range 6–25, SD: 3.64) in the nonviral group (*p* = 0.043). At the end of the follow-up we had still 25 patients (41%) who needed topical treatment to control the elevated IOP.

A glaucoma demonstrated by OCT and visual fields occurred in 15 patients (24.59%) (2: Fuchs, 1: VKH, 1: sarcoidosis, 4: CMV, 3: herpes, and 4: idiopathic). There were 8 cases of glaucoma found in the viral group (34.78%) and 7 cases of glaucoma found in the nonviral group (23.33%) (*p* = 0.774) (Table 3). Suspected but uncertain glaucoma was found in 11 patients (18.03%) (3 in the viral group and 8 in the nonviral group) (*p* = 0.401). No glaucoma was found in 31 patients of the whole group 14 viral and 21 nonviral (*p* = 0.774).

IOP could be successfully lowered under 25 mmHg in most patients (90.16%). No differences were found between groups (97.2% and 84% *p* = 0.149) (Figure 2).

Fifteen patients (25%) underwent surgery for uncontrolled IOP, 9 patients (25%) in the nonviral group and 6 patients (24%) in the viral group (*p* = 1.0). The etiologies of those patients were 1: Fuchs, 2: VKH, 1: sarcoidosis, 3: herpes, 2: CMV, 4: idiopathic, 1: Behçet, and 1: ankylosing spondylitis.

## 4. Discussion

Elevated IOP in uveitis does not always lead to glaucomatous damage; however glaucoma is one of the most severe complications of uveitis. The aim of this work was to evaluate the characteristics of uveitis with elevated IOP and the evolution toward glaucoma and to compare viral and nonviral hypertensive uveitis.

In order to study the evolution patients with elevated IOP since the beginning of the disease it was important to include only patients who had a complete follow-up since first attack and exclude patients that were already treated for uveitis before the referral in our department. This approach might explain some particularities of our cohort. Most patients with JIA were addressed to our referral center from another clinic; consequently only one patient with JIA from our group of patients with JIA and elevated IOP could be included in the study. This explains the low rate of JIA in our study. However when we analyze our complete cohort of patients with JIA referred later in the evolution of their uveitis to our clinic, 55% of them presented with a hypertony and 35% of them had a glaucoma (unpublished results). These results are more in accordance with other reports where a frequency from 14 to 42% of glaucoma was found in JIA [20–27]. The distribution of the other causes of uveitis is in agreement with previous reports [28]. We found 22.95% of anterior herpetic uveitis. This is slightly less than other reports where 28 to 45% of elevated IOP was found [28, 29].

Another limit of our study is that only 18/25 patients of the viral group had a PCR performed and a positive result for a virus. However the 7 patients without PCR had pathognomonic signs of herpetic anterior uveitis or Fuchs heterochromic anterior uveitis. Only 2 patients with a clinical diagnosis of Fuchs heterochromic uveitis had a positive PCR and none had a Goldman Witmer test performed for rubella virus. Wensig et al. found a positive Goldman Witmer ratio for rubella virus in 100% of patients with a clinical diagnosis of Fuchs while only 12% of these patients had a positive PCR for rubella virus [6].

To our knowledge, our study is the first one to evaluate the etiologies of uveitis among a group of hypertensive uveitis and to compare viral and nonviral uveitis, while most previous studies investigated elevated IOP among groups of patients with a specific etiology of uveitis [6, 10, 20, 21, 27, 28]. We found demographic differences between viral and nonviral uveitis. The patients of the viral group were older than the patients in the nonviral group. Indeed, the mean age for viral uveitis was 50.6 years while it was 34.7 years for nonviral causes. We did not compare the age among viral causes like Wensing et al., who compared RV with HSV and VZV and found that RV appeared in younger patients compared to HSV and VZV [6] while Miserocchi et al. also reported that the age was similar for HSV and VZV [20].

A major issue of hypertensive uveitis is to evaluate the relative role or inflammation and corticosteroid-response in the elevation of IOP. In this series, 30 patients (49.2%) had elevated IOP before the use of topical steroids drops. This means that, at least, half of the uveitis with elevated IOP were not related to the use of topical corticosteroid. Among these patients, there was no significant difference in IOP between viral and nonviral group. When all the 61 patients were evaluated, the mean value of the first elevated IOP and the mean highest value of IOP appeared to be significantly higher in the group of patients with a viral uveitis as compared with the patients with nonviral uveitis. However these values were not significant anymore when limited to the measurements of the 30 patients with elevated IOP before the use of topical corticosteroids. This might indicate that patients with viral and nonviral uveitis have comparable IOP or that the series limited to 30 patients was too little to show significant differences.

To our knowledge this is the first evaluation of initial elevated IOP in uveitis. Very few authors previously reported intraocular pressure values; most of them defined a limit beyond which the uveitis is defined as hypertensive but did not analyze IOP values. Values of high IOP have been reported recently in CMV uveitis; Park et al. reported an average maximal IOP of 33.9 in CMV uveitis which is similar to our values before corticosteroids (32.75) while the average maximal IOP was 47.3 mmHg among all our group of patients of CMV uveitis [8].

Our results in Fuchs uveitis with an average IOP of 36.32 mmHg at baseline and 40.28 mmHg as average maximal IOP with one patient who had a filtering surgery (16.67%) support the study of Bouchenaki and Herbort who reported a lower rate of filtering surgery in Fuchs uveitis compared with the other etiologies of uveitis. Unfortunately we cannot compare our values with their IOP values because we only analyzed the hypertensive uveitis and not the hypertensive uveitis among all uveitis cases [28, 30].

IOP could be successfully lowered under 25 mmHg in most patients in both groups (97.2% and 84%) and 24.6% of the patients underwent a glaucoma surgery (9 in the nonviral group and 6 in the viral group). This can be compared to the results of the study of Pogorzalek et al. where 25.9% of patients with elevated IOP underwent a surgery [28].

The glaucoma surgery rate varies among studies: Sungur et al. found only 2.6% (2 patients) of surgeries among viral hypertensive uveitis cases but this study was evaluating all patients with and without hypertensive uveitis, and their patients had herpetic keratouveitis rather than anterior uveitis without active keratitis. And if we report their 2 patients among their hypertensive cases, the rate of surgeries becomes 5.56% [21]. Sallam et al. found 14.5% of surgeries among elevated IOP uveitis cases [31].

A clear glaucoma occurred in 24.6% of our patients with hypertensive uveitis without significant differences between viral and nonviral uveitis. This confirms the study of Pogorzalek et al., while Panek et al. found 20% of glaucoma [27, 28]. Wensing et al. found 18 to 30% of glaucoma in viral uveitis with a lower rate of glaucoma in RV with clinical signs of Fuchs uveitis compared to in herpetic uveitis [6]. Sungur et al. found 13.1% of glaucoma in viral uveitis, but again this was a group of keratouveitis [21]. Our percentage is slightly higher but this can be explained by the design of our study evaluating only hypertensive uveitis which increased the risk to develop glaucoma compared with a group of uveitis including nonhypertensive as well as hypertensive uveitis.

Detection of glaucoma may be influenced by uveitic changes, uveitis may affect the visual field and inflammatory optic disc swelling may also obscure the assessment of glaucomatous optic disc. Therefore, as recently suggested by Din et al., OCT of the optic nerve had been preferentially evaluated for glaucomatous retinal RNFL changes in our patients with uveitis during quiescent periods to reduce the masking effect of RNFL thickening associated with active uveitis [32].

Although the two groups were comparable concerning the gender, the length of follow-up, and the number of patients, the two groups were not totally comparable since the age of patients was significantly different with 35.7 years for the nonviral group and 50.6 years in the viral group (*p* = 0.014) but this appears to be a characteristic of viral uveitis [19]. On the other side, some authors found younger patient in Fuchs uveitis and also in CMV uveitis while lymphoma and sarcoidosis are especially known to occur in older patients [6, 8, 32, 33].

## 5. Conclusion

This retrospective study evaluates the evolution of IOP in hypertensive uveitis and provides in addition a comparative approach of viral and nonviral hypertensive uveitis. We found an older age and a higher number of unilateral cases in viral hypertensive uveitis compared with nonviral cases. Half of the patients had the first episode of HIOP before the use of corticosteroids. A higher initial and maximal elevated IOP value in viral hypertensive uveitis compared with nonviral cases might also be possible. The risk to develop glaucoma in hypertensive uveitis is important and comparable in both groups. Prospective studies are needed to further validate these different characteristics.

## Figures and Tables

**Table 1:** Etiologies of uveitis.

Viral uveitis (*n* = 25)	Viral (*n* = 25)
	Fuchs (*n* = 5)
	Herpes (*n* = 14)
	Cytomegalovirus (*n* = 6)
	PCR+ (*n* = 18)
	Rubeola virus (*n* = 2)
	HSV1 (*n* = 10)
	Cytomegalovirus (*n* = 6)

Nonviral uveitis (*n* = 36)	Toxoplasmosis (*n* = 6)
	Behçet (*n* = 3)
	VKH (*n* = 3)
	AJI (*n* = 1)
	Idiopathic (*n* = 14)
	SPA (*n* = 2)
	Sarcoidosis (*n* = 7)

**Table 2:** Demographic characteristics of patients in viral and nonviral group.

	All patients	Viral	Nonviral	*p*
Number of patients	61	25	36	0.159^*∗*^
Age (years)	44	50.6	35.7	0.014^*∗∗*^
M/F	35/29	13/12	19/17	1^*∗∗∗*^
Follow-up (months)	67.8	65.9	69.1	0.74

^*∗*^Chi-squared, ^*∗∗*^ *t*-test for equal variance, ^*∗∗∗*^Fisher exact test.

**Table 3:** Laterality, IOP, and glaucoma.

	All patients (*n* = 61)	Viral (*n* = 25)	Nonviral (*n* = 36)	*p*
Bilaterality	16.39%	**n** = 0**(0%)**	27.78%	0.004
Time to EIOP (months)	9.28	9.84	8.89	0.08
First elevated IOP (mmHg)	32.95	36	27.5	0.008
Maximum IOP (mmHg)	36.6	40.28	34.06	0.015
Glaucoma	24.59%	32.00%	19.44%	0.774
